# Cause-specific mortality in the Kombewa health and demographic surveillance systems site, rural Western Kenya from 2011–2015

**DOI:** 10.1080/16549716.2018.1442959

**Published:** 2018-03-05

**Authors:** Peter Sifuna, Lucas Otieno, Sheila Ogwang, Bernhards Ogutu, Ben Andagalu, John Owuoth, Valentine Singoei, Jessica Cowden, Walter Otieno

**Affiliations:** ^a^ US Army Medical Research Directorate–Kenya (USAMRD-K)/Kenya Medical Research Institute (KEMRI), Kisumu, Kenya; ^b^ INDEPTH Network, Accra, Ghana; ^c^ Defense Institute for Medical Operations, TX, USA

**Keywords:** Verbal autopsy, InterVA-4, cause-specific mortality fraction, rural Kenya, INDEPTH network

## Abstract

**Background**: The vast majority of deaths in the health and Kombewa demographic surveillance system (HDSS) study area are not registered and reported through official systems of vital registration. As a result, few data are available regarding causes of death in this population.

**Objectives**: To describe causes of death among residents of all ages in the Kombewa HDSS, located in rural Western Kenya.

**Methods**: Verbal autopsy (VA) interviews at the site were conducted using the modified 2007 and later 2012 standardized WHO questionnaires. Assignment of causes of death was made using the InterVA-4 model version 4.02. Cox regression model, adjusted for sex, was built to evaluate the influence of age on mortality.

**Results**: There were a total of 5196 deaths recorded between 2011 and 2015 at the site. VA interviews were successfully completed for 3903 of these deaths (75.1%). Mortality rates were highest among neonates HR = 38.54 (<0.001) and among Infants HR = 2.07 (<0.006) in the Kombewa HDSS. Among those deaths in which VA was performed, the top causes of death were HIV/AIDS (12.6%), Malaria (10.3%), Pneumonia (10.1%), Acute abdomen (7.0%), Stroke (5.2%) and TB (4.9%) for the whole population in general. Stroke, acute abdomen heart diseases and Pneumonia were common causes of death (CODs) among the elderly over the age of 65.

**Conclusions**: The analysis established the main CODs among people of all ages within the area served by the Kombewa HDSS. We hope that information generated from this study will help better address preventable deaths in the surveyed community as well as help mitigate negative health impacts in other rural communities throughout the Western Kenya region.

## Background

Reliable and timely cause specific mortality data form the basis for health care planning, resource allocation and evaluating impact of interventions [1–3]. Conventionally, health facilities play a predominant role in generating and compiling local health information. However, in most developing countries, health care utilization is generally low with huge discrepancies between different social groups [4]. In such settings, the majority of deaths occur at home and vital registration systems are often incomplete or nonexistent [5]. Lack of quality health care data may cause local health care problems to be misjudged, thereby making it difficult to formulate optimal health care policies. One solution to address the incomplete nature of local health care facility data is to collect prospective data from a population as is done in a health and demographic surveillance system (HDSS).

A HDSS longitudinally monitors births, deaths, causes of death, migration, and other health and socio-economic indicators within a defined population. These data are useful in providing data complementary to facility-based reporting systems and supplementary to large scale surveys (such as census surveys), which are usually conducted after long intervals [6]. In such settings, verbal autopsy (VA) methods are increasingly being used to derive population cause of death data [7]. VA is a useful tool for establishing the probable cause of death (COD) by interviewing a caregiver or a close person who witnessed the death event [1]. The usefulness of the VA tool for filling the information gap on causes of death in low and middle income countries has been discussed extensively [7–10]. In the present study, we describe the main causes of death in the Kombewa HDSS, in rural Western Kenya, in the years 2011–2015. Information on the sex- and age-specific causes of death were determined by VA; these provide important insight into public health issues and the epidemiology of disease in this cohort of nearly 141,956 people living in rural Western Kenya. To the best of our knowledge, there is no cause-specific mortality data available for the surveyed population. This information may help better address preventable deaths in the surveyed communities as well as help mitigate negative health impacts in other rural communities throughout the Western Kenya region.

## Methods

### Study setting

Data analyzed for this study were collected under the Kombewa HDSS, run by the KEMRI/Walter Reed Project in a rural portion of Kisumu County, Western Kenya. Details of the Kombewa HDSS catchment area have been described in detail elsewhere [11]. In brief, the Kombewa HDSS covers an area of about 369 km^2^ along the north-eastern shores of Lake Victoria.

**Figure 1.**
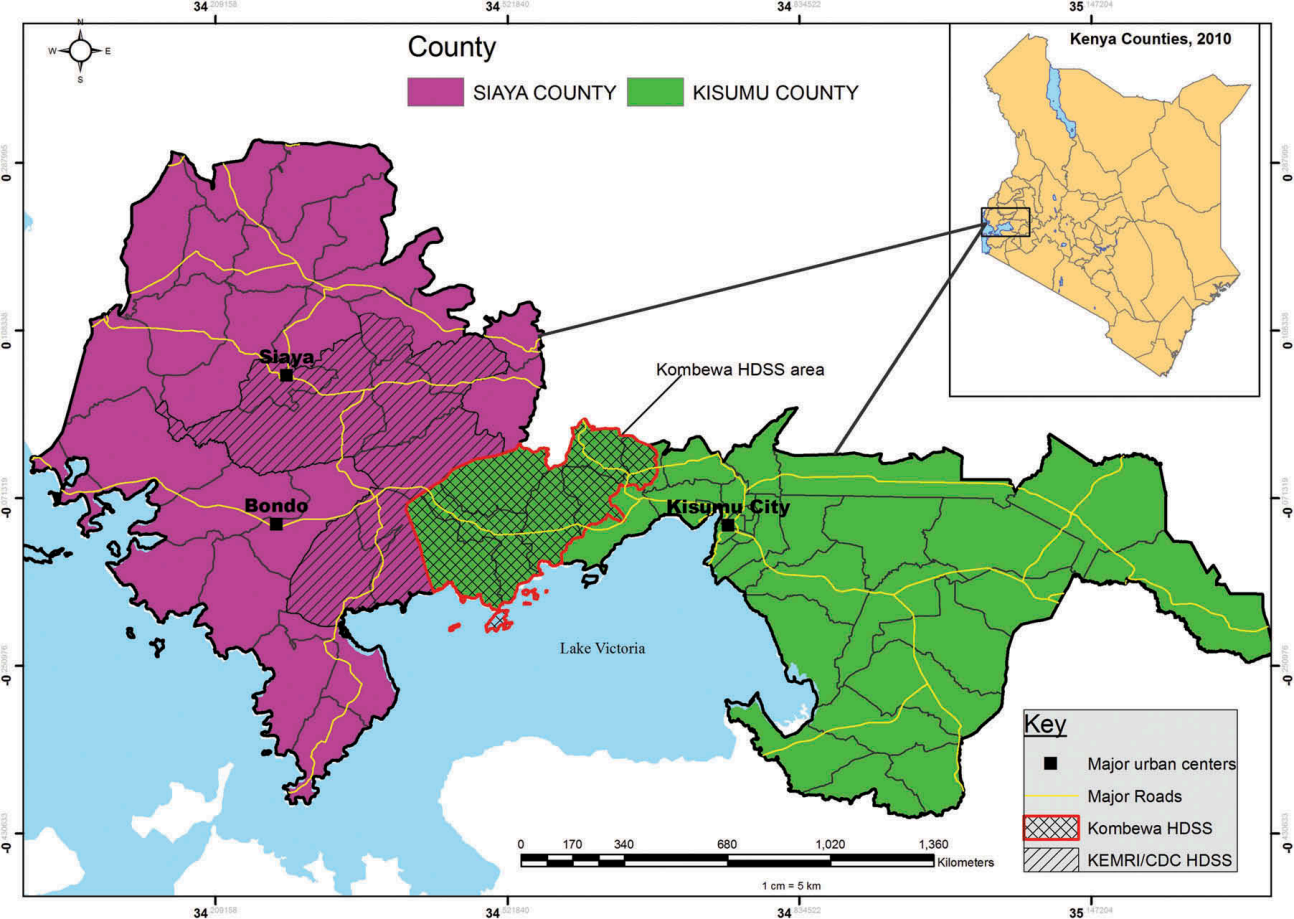
Location of the Kombewa HDSS catchment area, boarded by KEMRI/CDC-Kisumu HDSS to the west.

The HDSS is located about 40 km West of Kisumu City, which is the administrative capital of Kisumu County. It boarders the KEMRI/CDC HDSS to the west, in the neighboring Siaya County (Figure 1). A dynamic cohort of 141,956 individuals drawn from 34,718 households forms the HDSS surveillance population. Following a baseline survey conducted in 2011, the HDSS continues to monitor key population changes through bi-annual household surveys. Routine surveys capture information on individuals and households, including residency, household relationships, births, deaths, migrations (in and out), causes of morbidity (syndromic incidence and prevalence), and causes of death by way of verbal autopsy. The HDSS population is characterized by low economic status and high prevalence of infectious diseases such as TB, HIV, and Malaria [12–14]. The HIV prevalence in the region encompassing the entire HDSS is nearly four times the nationally reported average, with a general prevalence in Kisumu County of 20% as compared to the national prevalence of 5.1% [14]. The burden of Tuberculosis (TB) disease is estimated at 500–600 cases per 100,000 [13].

### Data collection methods

Deaths of HDSS residents were collected routinely by trained field workers during biannual house visits. Residents in the HDSS are defined as all persons residing in the study area for 6 months or more, excluding transient residents and visitors. In addition to the routine house visits, a team of dedicated ‘village reporters,’ largely drawn from a pool of Ministry of Health (MOH) trained Community Health Volunteers (CHVs), provided death notification within 7 days of an event. The notified events were thereafter verified and registered into the database by a team of HDSS field staff. Recorded deaths were then followed up with a standardized VA interview by specially trained lay interviewers to record events surrounding death. VA interviews were conducted using the modified 2007 then the 2012 standardized WHO questionnaires recommended by INDEPTH for deaths occurring in the HDSS [15,16]. The VA tool consists of three separate VA questionnaires used to collect data for neonates (0–28 days old), children (29 days–14 years old), and adolescents and adults (15+ years). The questionnaires contain a short open history section followed by a series of closed questions. The open history portion provides the respondent’s chronological account of illnesses and events leading to death, while the closed questions filter through the history and details of the illness. VA interviews were performed a few weeks after burial to respect the mourning period, while still facilitating recall. Absence of an adult in the home was recorded as ‘no respondent.’

### Assignment of cause of death

Assignment of cause of death (COD) was made using the InterVA-4 model version 4.02. A detailed description of the InterVA-4 model has been given elsewhere [17]. In brief, the InterVA-4 model is a computer based logarithm which uses Bayes’ theorem in an attempt to overcome limitations of alternative methods [17–20], such as physician coding. InterVA-4 ascertains probable cause(s) of death for each VA case based on expert algorithms and relevant available medical data [21]. The causes of death generated by the InterVA-4 are compatible with the International Classification of Diseases version 10 (ICD-10) and are categorized into 62 groups, as defined in the 2012 WHO VA instrument [16]. VA data obtained at the site prior to the WHO 2012 standards were retrospectively transformed into the WHO 2012 and InterVA-4 input format for processing. The model requires a local setting for the prevalence of malaria and HIV in the population [22]; Malaria and HIV prevalence were both set to ‘high’ for all age categories for the Kombewa HDSS.

### Statistical analyses

Cause-specific mortality rates were calculated as the number of cause-specific deaths per 1000 person-years of follow-up and per 1000 live births (in the case of infant and under five mortality rates). The Cox regression model, adjusted for sex, was built to evaluate the influence of age on mortality. Annual mortality rates over the five year period were also calculated for the assessment of cause-specific mortality trends. P-value of <0.05 was considered significant. All analyses were performed using STATA version 12 (Stata Corporation, College Station, TX, USA).

## Results

### Crude mortality

The analysis presented in this paper is based on follow-up of 650 919.2 person-years during 2011–2015. There were a total of 5196 registered deaths between 2011 and 2015 at the site. For 3903 of these deaths (75.1%) VA interviews were successfully completed. Among those deaths in which a VA was performed, 62.1% occurred at home and 37.9% occurred in a health facility. Most neonatal deaths (63.2%) occurred in hospitals while more of the elderly died at home (71.7%). The crude death rate for the site was 8.0 per 1000 person years with higher rates for males [Mortality rate ratio (95% CI):1.20 (1.14–1.27)]. Table 1 summarizes the mortality rates for the different age groups by sex. The percentages of VA interviews conducted for the different age groups are also summarized in Table 1.

**Table 1.** Number of deaths, proportion of deaths with verbal autopsy and death rates by age and sex.

Total deaths			% of deaths with VA		Mortality rates					
Age Group	Male	Female	Male	Female	Male	Female	Total	Rate ratio (95% CI)	Hazard ratio^a^	P-value
Infants	198	194	148 (74.7)	141 (72.7)	36.8	33.9	35.3^b^	1.08 (0.89–1.33)	2.42	<0.001
Under 5	464	476	396 (85.3)	402 (84.5)	86.3	83.3	84.7^c^	1.04 (0.91–1.18)	1^e^	–
5–14.	170	136	112 (65.9)	95 (69.9)	1.7	1.4	1.5^d^	1.26 (1.00–1.59)	0.80	0.003
15–49	835	647	622 (74.5)	470 (72.6)	6.5	4.4	5.3^d^	1.49 (1.34–1.65)	0.68	<0.001
50–64	376	322	311 (82.7)	235 (73)	19.1	10.4	13.8^d^	1.83 (1.57–2.13)	0.66	<0.001
65+	832	938	672 (80.8)	691 (73.7)	61.7	46.4	52.5^d^	1.33 (1.21–1.46)	0.57	<0.001
Total	**2677**	**2519**	**2063 (77.1)**	**1840 (73)**	**8.8**	**7.3**	**8.0**	**1.20 (1.14–1.27)**	**_**	**_**

^a^ Hazard ratio = Cox regression, adjusted.

^b^ Infant mortality = Infant deaths per 1000 live births.

^c^ Under 5 mortality = Under 5 deaths per 1000 live births.

^d^ Rate based on person-time contributed by each age category.

^e^ Reference age group.

**Figure 2.**
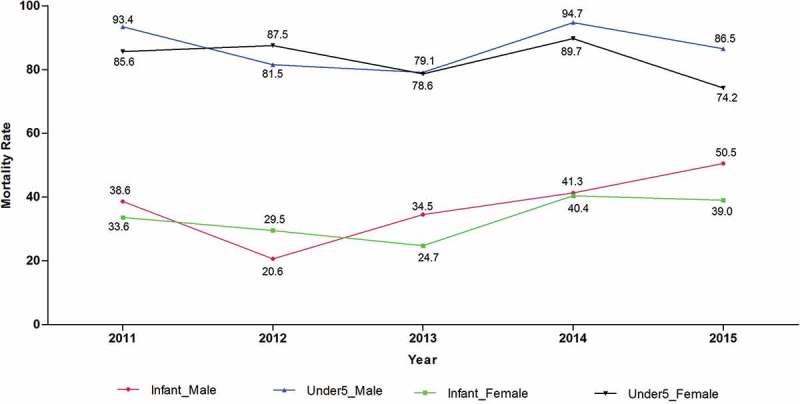
Infant and under five mortality rates for the Kombewa, 2011–2015.

Overall, 11,093 live births were registered between 2011 and 2015 in the Kombewa HDSS (5377 boys and 5716 girls). In the same period, the overall infant mortality rate (defined as the number of all cause-unspecific deaths of newborns within the first year after birth per 1000 live. births) was 35.0 deaths per 1000 live births. The overall under five mortality rate (defined as the number of all cause-unspecific deaths of children under the age of five per 1000 live. births) was 85.0 deaths per 1000 live births. The infant mortality rate and under five mortality rate for the site over the five year period are presented below (Figure 2).

### Cause-specific mortality as determined by InterVA-4

The top causes of death for the 3903 deaths (75.1%) with VA interviews were HIV/AIDS (12.6%), Malaria (10.3%), pneumonia (10.1%), acute abdomen (7.0%), stroke (5.2%), and pulmonary Tuberculosis (PTB), 4.9%. Deaths that could not be assigned a cause of death (indeterminate) translated to 12.9% of all deaths. Table 2 presents hazard ratios for the top causes of death by age.

**Table 2.** Top 10 causes of death by age group.

	Age group						
Cause of death	Neonate	Infant	1–4*	5–14	15–49	50–64	65+
HIV/AIDS	–	2.39 (<0.001)	1	0.75 (0.070)	0.73 (0.006)	0.61 (<0.001)	0.52 (<0.001)
Malaria	18.33 (<0.001)	2.80 (<0.001)	1	0.65 (0.002)	0.74 (0.029)	0.61 (0.017)	0.49 (<0.001)
Pneumonia	–	1.80 (0.001)	1	0.78 (0.267)	0.54 (<0.001)	0.62 (0.013)	0.41 (<0.001)
Acute abdomen	–	1.19 (0.459)	1	0.49 (0.015)	0.71 (0.849)	0.58 (0.156)	0.63 (0.048)
Stroke	–	–	1	–	0.37 (<0.001)	0.35(<0.001)	0.24 (<0.001)
PTB	–	–	1	2.29 (0.002)	0.94 (0.761)	1.18 (0.531)	0.76 (0.173)
Digestive neoplasms	–	–	1	–	1.53 (0.150)	1.25 (0.263)	–
Cardiac diseases	–	–	1	–	1.85 (0.035)	0.98 (0.949)	–
Diabetes	–	–	1	2.78 (0.012)	2.01 (0.038)	1.70 (0.184)	1.04 (0.909)
Diarrhea	–	1.64 (0.008)	1		0.80 (0.662)	1.11 (0.632)	0.71 (0.161)
Indeterminate	33.63 (<0.001)	3.060 (<0.001)	1	1.12 (0.652)	0.72 (0.078)	0.59 (0.012)	0.61 (0.007)
**All causes**	**38.54 (<0.001)**	**2.07 (<0.006)**	**1**	**0.79 (<0.001)**	**0.68 (<0.001)**	**0.65 (<0.001)**	**0.56 (<0.001)**

1–4* = Reference point.

Other top causes of death in the Kombewa HDSS included stomach cancers (4.0%), cardiac diseases (3.1%), unspecified cancers (2.2%) and diarrhea (2.2%). Table 3 presents all cause-specific mortality fractions by age group as assigned by the InterVA-4 model.

**Table 3.** Cause-specific mortality fractions by age group as assigned by the InterVA-4 model.

Cause of death and ICD-10 codes	Neonate	Infant	1–4	5–14	15–49	50–64	65+	Total
01.01 Sepsis (non-obstetric)	0.0	0.0	0.0	0.0	0.0	0.0	0.1	**0.2**
01.02 Acute resp infect incl. pneumonia	0.0	1.6	1.8	0.8	2.2	1.3	2.5	**10.1**
01.03 HIV/AIDS related death	0.0	0.6	1.4	0.6	6.6	2.0	1.4	**12.6**
01.04 Diarrhoeal diseases	0.0	0.5	0.9	0.1	0.2	0.1	0.4	**2.2**
01.05 Malaria	0.0	1.4	3.9	1.5	1.7	0.6	1.2	**10.3**
01.06 Measles	0.0	0.2	0.2	0.0	0.0	0.0	0.0	**0.4**
01.07 Meningitis and encephalitis	0.0	0.1	0.1	0.2	0.8	0.2	0.3	**1.6**
01.09 Pulmonary tuberculosis	0.0	0.0	0.1	0.2	2.2	0.8	1.8	**4.9**
01.10 Pertussis	0.0	0.0	0.0	0.0	0.0	0.0	0.0	**0.0**
01.99 Other and unspecified infect. dis.	0.0	0.0	0.0	0.0	0.3	0.2	0.4	**0.9**
02.01 Oral neoplasms	0.0	0.0	0.0	0.0	0.2	0.1	0.2	**0.5**
02.02 Digestive neoplasms	0.0	0.0	0.0	0.0	0.7	1.2	2.1	**4.0**
02.03 Respiratory neoplasms	0.0	0.0	0.0	0.0	0.1	0.2	0.4	**0.7**
02.04 Breast neoplasms	0.0	0.0	0.0	0.0	0.2	0.0	0.0	**0.2**
02.05 & 02.06 Reproductive neoplasms	0.0	0.0	0.0	0.0	0.2	0.1	1.0	**1.3**
02.99 Other and unspecified neoplasms	0.0	0.0	0.0	0.0	0.3	0.5	1.4	**2.2**
03.01 Severe anaemia	0.0	0.0	0.0	0.0	0.1	0.1	0.3	**0.4**
03.02 Severe malnutrition	0.0	0.0	0.2	0.1	0.2	0.1	0.6	**1.1**
03.03 Diabetes mellitus	0.0	0.0	0.1	0.0	0.3	0.5	1.3	**2.2**
04.01 Acute cardiac disease	0.0	0.0	0.0	0.0	0.4	0.4	1.1	**1.9**
04.02 Stroke	0.0	0.0	0.0	0.0	0.7	1.1	3.4	**5.2**
04.03 Sickle cell with crisis	0.0	0.1	0.1	0.2	0.0	0.0	0.0	**0.4**
04.99 Other and unspecified cardiac dis.	0.0	0.0	0.0	0.0	0.4	0.2	2.5	**3.1**
05.01 Chronic obstructive pulmonary dis.	0.0	0.0	0.0	0.0	0.0	0.1	0.9	**0.9**
05.02 Asthma	0.0	0.0	0.1	0.1	0.1	0.1	0.1	**0.4**
06.01 Acute abdomen	0.0	0.2	0.1	0.3	1.7	1.4	3.3	**7.0**
06.02 Liver cirrhosis	0.0	0.0	0.0	0.0	0.2	0.1	0.5	**0.8**
07.01 Renal failure	0.0	0.0	0.1	0.0	0.1	0.3	0.7	**1.2**
08.01 Epilepsy	0.0	0.1	0.0	0.1	0.4	0.1	0.2	**0.7**
09.01 Ectopic pregnancy	0.0	0.0	0.0	0.0	0.0	0.0	0.0	**0.0**
09.02 Abortion-related death	0.0	0.0	0.0	0.0	0.1	0.0	0.0	**0.1**
09.03 Pregnancy-induced hypertension	0.0	0.0	0.0	0.0	0.1	0.0	0.0	**0.1**
09.04 Obstetric haemorrhage	0.0	0.0	0.0	0.0	0.1	0.0	0.0	**0.1**
09.06 Pregnancy-related sepsis	0.0	0.0	0.0	0.0	0.2	0.0	0.0	**0.2**
09.07 Anaemia of pregnancy	0.0	0.0	0.0	0.0	0.0	0.0	0.0	**0.0**
09.99 Unspecified maternal CoD	0.0	0.0	0.0	0.0	0.1	0.0	0.0	**0.1**
10.01 Prematurity	0.3	0.0	0.0	0.0	0.0	0.0	0.0	**0.3**
10.02 Birth asphyxia	0.4	0.0	0.0	0.0	0.0	0.0	0.0	**0.4**
10.03 Neonatal pneumonia	0.2	0.0	0.0	0.0	0.0	0.0	0.0	**0.3**
10.04 Neonatal sepsis	0.0	0.0	0.0	0.0	0.0	0.0	0.0	**0.0**
10.06 Congenital malformation	0.1	0.1	0.0	0.0	0.0	0.0	0.0	**0.1**
10.99 Unspecified neonatal CoD	0.4	0.0	0.0	0.0	0.0	0.0	0.0	**0.4**
11.02 Macerated stillbirth	0.0	0.0	0.0	0.0	0.0	0.0	0.0	**0.0**
12.01 Road traffic accident	0.0	0.1	0.0	0.2	0.7	0.2	0.5	**1.7**
12.02 Other transport accident	0.0	0.0	0.1	0.1	0.4	0.1	0.3	**1.0**
12.03 Accid fall	0.0	0.0	0.0	0.0	0.0	0.1	0.2	**0.3**
12.04 Accid drowning and submersion	0.0	0.0	0.0	0.1	0.2	0.1	0.1	**0.5**
12.05 Accid expos. to smoke fire & flame	0.0	0.0	0.1	0.0	0.1	0.1	0.1	**0.3**
12.06 Contact with venomous animal	0.0	0.0	0.1	0.1	0.1	0.1	0.1	**0.3**
12.07 Accid poisoning & noxious subs.	0.0	0.0	0.1	0.0	0.0	0.0	0.0	**0.1**
12.08 Intentional self-harm	0.0	0.0	0.0	0.1	0.9	0.2	0.3	**1.6**
12.09 Assault	0.0	0.0	0.1	0.0	0.8	0.2	0.1	**1.2**
12.99 Unspecified external CoD	0.0	0.0	0.0	0.0	0.1	0.1	0.1	**0.4**
98 Other and unspecified NCD	0.0	0.0	0.0	0.0	0.1	0.0	0.3	**0.4**
99 Cause of death unknown	0.4	0.6	1.1	0.5	3.9	1.4	5.0	**12.9**
**All causes**	**1.7**	**5.5**	**10.6**	**5.3**	**28.0**	**14.0**	**34.9**	**100.0**

**Figure 3.**
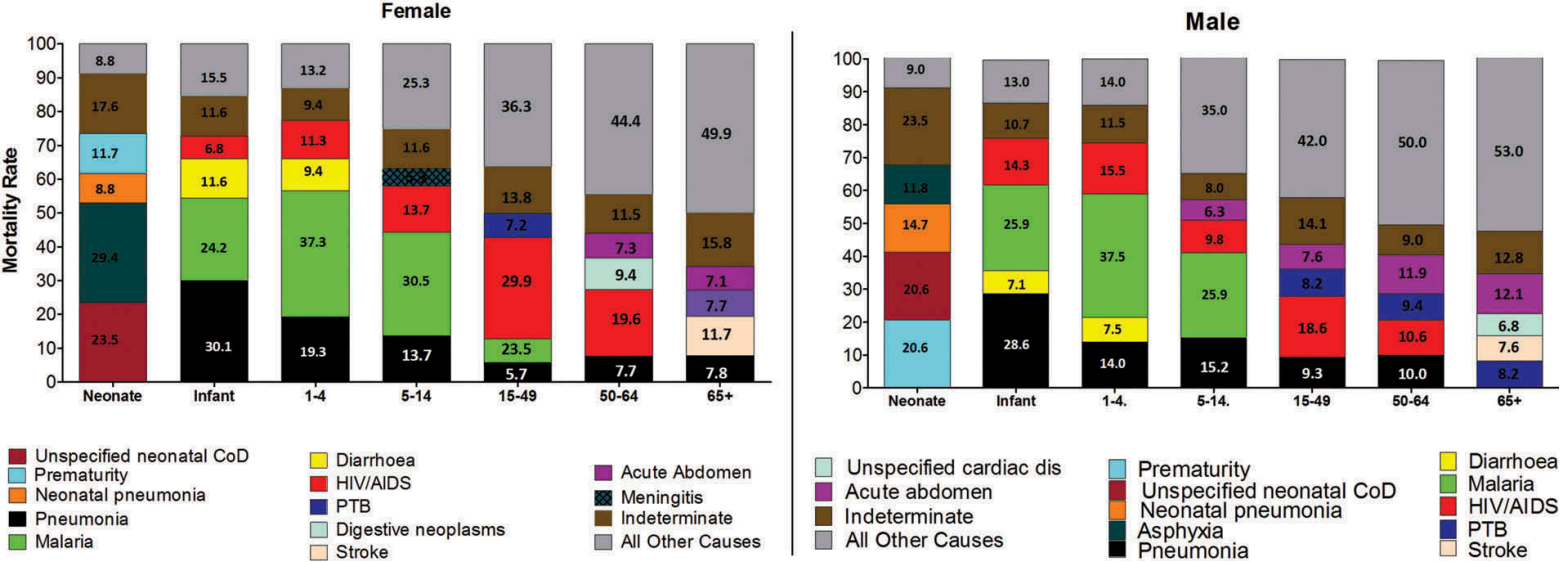
Top five mortality fractions by age group and sex. Figure 3. The top five cause-specific mortality fractions among each age group by sex as derived by the InterVA-4 model.

**Figure 4.**
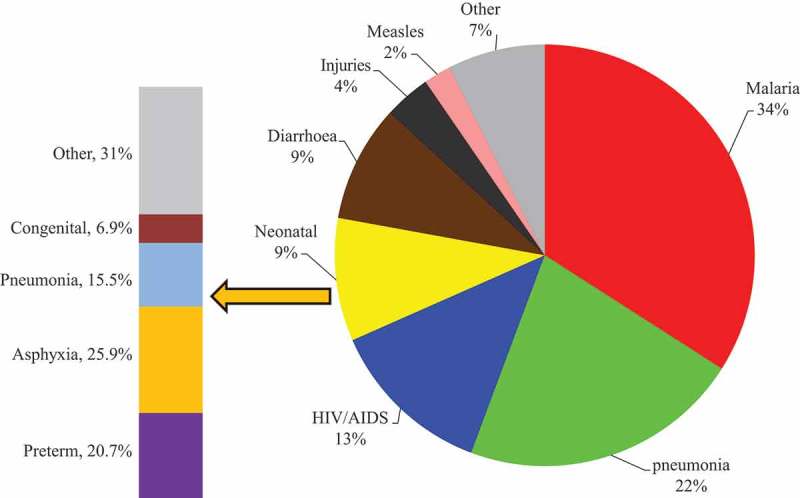
The top causes of mortality fractions among children under five years as derived by the InterVA-4 model.

Among neonates (0–28 days), unspecified neonatal causes (22.1%), birth asphyxia (20.6%), prematurity (16.2%), neonatal pneumonia (11.8%), and congenital malformation (2.9%) were the top causes of death. Preterm deaths accounted for 20.6% of female deaths and 11.8% of male deaths in this age group. The leading causes of death among neonates (0–28 days) are presented in Figures 3 and 4. Indeterminate deaths accounted for 20.6%,HR = 33.63 (<0.001), Table 1.

Infant mortality rate for the site was 35.3 per 1000 live births. The infant mortality rate amongst females was 33.9 per 1000 live births and 36.8 per 1000 among males for this age category. The leading causes of death among infants outside of the neonatal period were pneumonia (29.3%), malaria (25.1%), HIV/AIDS (10.7%), diarrhea (9.3%), and measles (3.3%). The Hazard Ratio for communicable diseases (HIV/AIDS, pneumonia and malaria) was >1.80 for this age category, Table 1. (Indeterminate deaths accounted for 11.2% of infant deaths. The leading causes of death among infants are presented in Figures 3 and 4.

Malaria was the leading cause of death in children aged 1–4 years, accounting for 37.4% of all deaths with VA interviews in this category. The other top causes of death included pneumonia (16.7%), HIV/AIDS (13.3%), and diarrhea (8.5%). Pneumonia accounted for 19.3% of deaths among females and 14% of deaths in males in this age group. Indeterminate causes of death accounted for 10.4% in this age category. The leading causes of death among children under the age of 5 years are presented in Figures 3 and 4.

The overall mortality rate for the age group 5–14 years was 1.5 per 1000 p-y. Malaria was the leading cause of death (28.0%), followed by pneumonia (14.5%), HIV/AIDS (11.6%), acute abdomen 5.3%, and road traffic accidents (4.3%)for this age group. The rates of PTB and diabetes were significantly high in this age category (HR = 2.29 (0.002) and 2.78 (0.012), respectively). Indeterminate COD accounted for 9.7% of deaths for this age group. The leading causes of death among the age group 5–14 years are presented in Figure 3.

The overall mortality rate for the age group 15–49 years was 5.3 per 1000 p-y. This age group accounted for 28.0% of all deaths for which VAs were available. HIV/AIDS was the leading cause of death (23.5%), followed by pneumonia (7.8%), PTB (7.8%), acute abdomen (6.1%), and malaria (5.9%) among persons aged 15–49 years. NCDs such as stomach cancers [HR = 1.53 (0.130)], cardiac diseases [HR = 1.85 (0.035)], and diabetes [HR = 2.01 (0.038)] were significantly higher in this age category, Table 1. Indeterminate causes accounted for 14.0% of VAs completed in this category. The leading causes of death among the age group 15–49 years are presented in Figure 3.

The overall mortality rate for the age group 50–64 years was 13.8 per 1000 p-y representing 14.0% of all deaths for which VA interviews were available. The lead cause of death among this age group was HIV/AIDS (14.5%), followed by acute abdomen (9.9%), pneumonia (9.0%), digestive neoplasm (8.3%), and stroke (7.5%). NCDs (stroke, cancers, cardiac diseases and diabetes) were significantly high in this age category (HR = 1.18–0.531), Table 1. Under CD, PTB and diarrhea were high among this age category, [HR = 1.18 (0.531) and HR = 1.11 (0.632), respectively], Table 1. Indeterminate causes accounted for 10.1% of VAs completed in this category. The leading causes of death among the age group 50–64 years are presented in Figure 3.

The overall mortality rate for the age group 65+ was 53 per 1000 p-y. The leading COD were stroke (9.7%), acute abdomen (9.5%), cardiac diseases (7.3%), pneumonia (7.1%), and stomach cancers (6.0%). Deaths due to diabetes were significantly higher in this age category [HR = 1.04 (0.909)], Table 1. Deaths due to PTB accounted for 8.2% male deaths and 2.3% female deaths in this age category. The leading causes of death among the age group 65+ years are presented in Figure 3.

The causes of death among children under the age of five (males and female) are presented below (Figure 4).

### Broad pattern of cause of death

**Figure 5.**
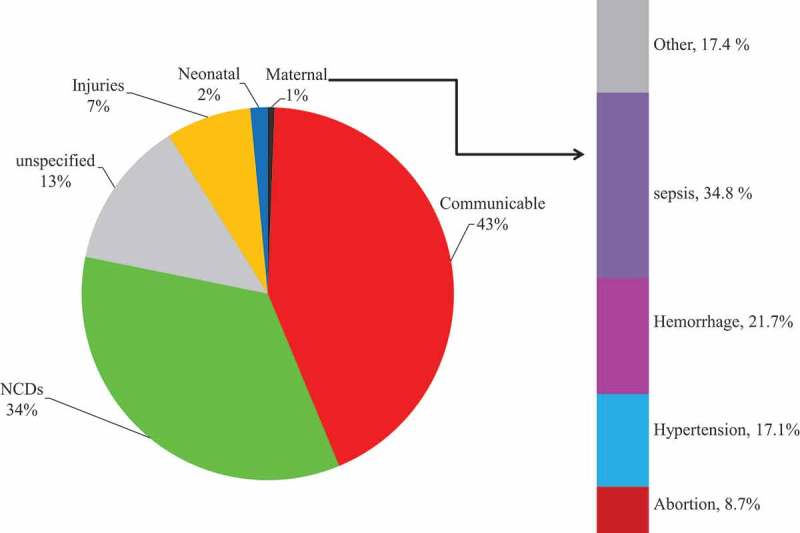
Broad causes of mortality fractions 2011–2015.

The specific causes were then broadly categorized into non-communicable diseases (NCDs), communicable diseases (CDs), neonatal causes, maternal causes, indeterminate, and external causes (injuries). When grouped according to broad causes, CDs were the leading cause of death overall (43%), followed by NCDs (34%), indeterminate (13%), external (7%), neonatal (2%), and maternal causes (1%) (Figure 5).

### Mortality due to CDs

CDs represented the most frequent cause of death in the Kombewa HDSS. Overall, 43.1% of the deaths subjected to InterVA-4 were due to CDs, resulting in a mortality rate of 2.6 deaths per 1000 person-years. Among infants, CDs accounted for 80.0% of all causes of infant deaths. The most prominent CDs for this age category were pneumonia, malaria and HIV (Table 2). CDs were also prominent among the age group 1–4 years (accounting for 79.4% of all causes of deaths) and 5–14 years (representing 62.8% of all causes of deaths). In the age group 15–49, CDs accounted for 49.6% of all causes of death. The prominent causes in the age group 15–49 category was HIV (23.5%), PTB (7.8%), and pneumonia (7.8%), Table 4.

**Table 4.** Broad causes fractions by age group as assigned by the InterVA-4 model.

		Age group						
Cause of death and ICD-10 codes	(N)	Neonate	Infant	1–4	5–14	15–49	50–64	65+
Communicable	**1684 (43.1)**	**2.9**	**80.0**	**79.4**	**62.8**	**49.6**	**36.9**	**22.7**
01.02 Acute resp. infect. incl. pneumonia	394 (10.1)	1.5	29.3	16.7	14.5	7.8	9.0	7.1
01.03 HIV/AIDS related death	491 (12.6)	0.0	10.7	13.3	11.6	23.5	14.5	3.9
01.04 Diarrhoeal diseases	84 (2.2)	0.0	9.3	8.5	1.0	0.8	0.7	1.0
01.05 Malaria	401 (10.3)	1.5	25.1	37.4	28.0	5.9	4.2	3.4
01.07 Meningitis and encephalitis	64 (1.6)	0.0	1.9	1.0	3.9	2.7	1.3	0.8
01.09 Pulmonary tuberculosis	193 (4.9)	0.0	0.0	0.5	2.9	7.8	5.5	5.1
Other and unspecified	57 (1.5)	0.0	3.7	1.9	1.0	1.0	1.7	1.4
NCDs	**1344 (34.4)**	**0.0**	**5.6**	**5.1**	**14.5**	**22.1**	**46.1**	**57.8**
02.02 Digestive neoplasms	156 (4)	0.0	0.0	0.0	0.0	2.7	8.3	6.0
03.02 Severe malnutrition	43 (1.1)	0.0	0.5	1.7	1.4	0.5	0.7	1.6
03.03 Diabetes mellitus	85 (2.2)	0.0	0.0	0.5	0.5	1.0	3.5	3.8
04.01 Acute cardiac disease	74 (1.9)	0.0	0.0	0.0	0.0	1.6	2.9	3.0
04.02 Stroke	202 (5.2)	0.0	0.0	0.0	0.5	2.6	7.5	9.7
04.99 Unspecified cardiac dis	122 (3.1)	0.0	0.0	0.0	0.0	1.3	1.7	7.3
06.01 Acute abdomen	272 (7)	0.0	3.3	0.7	5.3	6.1	9.9	9.5
07.01 Renal failure	46 (1.2)	0.0	0.0	0.5	0.0	0.5	1.8	2.1
08.01 Epilepsy	29 (0.7)	0.0	0.9	0.0	1.9	1.3	0.6	0.4
Other and unspecified	315 (8.1)	0.0	0.9	1.7	4.8	4.7	9.2	14.3
Neonatal	**59 (1.5)**	**76.5**	**1.9**	**0.5**	**0.5**	**0.0**	**0.0**	**0.0**
10.01 Prematurity	12 (0.3)	16.2	0.0	0.2	0.0	0.0	0.0	0.0
10.02 Birth asphyxia	15 (0.4)	20.6	0.5	0.0	0.0	0.0	0.0	0.0
10.03 Neonatal pneumonia	10 (0.3)	11.8	0.0	0.2	0.5	0.0	0.0	0.0
10.06 Congenital malformation	4 (0.1)	2.9	0.9	0.0	0.0	0.0	0.0	0.0
Other and unspecified	18 (0.5)	25.0	0.5	0.0	0.0	0.0	0.0	0.0
Maternal	**23 (0.6)**	**0.0**	**0.0**	**0.0**	**0.0**	**2.1**	**0.0**	**0.0**
09.03 Pregnancy-induced hypertension	4 (0.1)	0.0	0.0	0.0	0.0	0.4	0.0	0.0
09.04 Obstetric haemorrhage	5 (0.1)	0.0	0.0	0.0	0.0	0.5	0.0	0.0
09.06 Pregnancy-related sepsis	8 (0.2)	0.0	0.0	0.0	0.0	0.7	0.0	0.0
Other and unspecified	6 (0.2)	0.0	0.0	0.0	0.0	0.5	0.0	0.0
Injuries	**289 (7.4)**	**0.0**	**1.4**	**4.6**	**12.6**	**12.2**	**7.0**	**5.1**
12.01 Road traffic accident	68 (1.7)	0.0	0.9	0.0	4.3	2.6	1.7	1.5
12.02 Other transport accident	40 (1)	0.0	0.0	1.0	2.4	1.6	0.6	0.8
12.05 Accid expos to smoke fire & flame	13 (0.3)	0.0	0.5	0.5	0.0	0.5	0.4	0.2
12.06 Contact with venomous plant/animal	12 (0.3)	0.0	0.0	0.5	1.4	0.3	0.4	0.1
12.08 Intentional self-harm	61 (1.6)	0.0	0.0	0.0	2.4	3.2	1.5	1.0
12.09 Assault	48 (1.2)	0.0	0.0	1.0	0.5	3.0	1.1	0.3
12.99 Other and unspecified	47 (1.2)	0.0	0.0	1.7	1.4	1.1	1.5	1.2
Undetermined or unspecified	**504 (12.9)**	**20.6**	**11.2**	**10.4**	**9.7**	**14.0**	**10.1**	**14.3**
99 Cause of death unknown	504 (12.9)	20.6	11.2	10.4	9.7	14.0	10.1	14.3
All causes	**3903 (100.0)**	**100.0**	**100.0**	**100.0**	**100.0**	**100.0**	**100.0**	**100.0**

Table 4: Broad causes of mortality fractions by age group and sex as assigned by the InterVA-4 model.

### Mortality due to NCDs

NCDs accounted for 34.4% of all deaths, with an overall mortality rate of 2.1 deaths per 1000 person-years. NCDs were most prominent in the age groups 50–64 and 65+ years, accounting for 46.1 and 57.8% of all causes of death within these age categories, respectively. The most prominent causes of NCDs within the age groups 50–64 and 65+ years were stomach cancers, stroke, acute abdomen, and unspecified NCDS (Table 1).

### Mortality due to maternal and neonatal conditions

Deaths due to maternal and neonatal conditions accounted for 2.1% of all analyzed deaths. The main and typically neonatal conditions were unspecified neonatal causes (25.0%), asphyxia (20.6%), preterm (16.3%), and pneumonia (11.8%) of all examined neonatal deaths. The main causes of maternal deaths linked with pregnancy were sepsis, hemorrhage, and hypertension (Table 1). Maternal deaths were prominent among women aged 24–29 years of age, with that age cohort accounting for 26.9% of maternal deaths. Cumulatively, women between 14 and 30 years accounted for 56.5% of all maternal deaths that were subjected to VA.

### Mortality due to injuries

There were a total of 289 deaths (7.4%) due to injuries during the reporting period, with males accounting for 65.4% of deaths due to external causes. Death due to injuries was most prominent in the age groups 5–14 years (accounting for 12.6% of all causes of death in this age category) and in the age group 15–49 (12.2% of all causes of death in this age category). As summarized in Table 4, the most important sources of fatal trauma and injury were traffic accidents, suicide, and assault.

## Discussion

Mortality rates were highest among neonates HR = 38.54 (<0.001) and among infants HR = 2.07 (<0.006) in the Kombewa HDSS (Table 2). The cumulative infant mortality rate and under five mortality rate for site was 35.3 per 1000 live births and 84.7 per 1000 live births, respectively (see Table 1). When compared to the available national average data, the infant mortality rate from the HDSS was slightly lower than the national average of 39 per 1000 live births. When compared to the corresponding time period relating to the national data (2014), the infant mortality rates were almost similar between the HDDS (41 per 1000 live births) and DHS (39 per 1000 live births). The under five mortality rate for the HDSS (85 per 1000 live births) was, however, higher than the national average (52 per 1000 live births) [23]. When broken down by year, the infant mortality rate is lowest in 2011 and began to peak in 2014 (see Figure 2). It’s important to note that 2011 was the baseline year for the HDSS. Between 2011 and 2013, the program solely relied on biannual house visits to update data on births and deaths. Given the duration between each house visit (6 or more months), it is expected that some of the vital events such as neonatal and infant deaths would have been missed. In 2014 the program incorporated village reporters to assist in timely reporting of births and deaths, a likely reason for the steady increase in infant mortality rates from 2014 onwards.

In the population of the Kombewa HDSS, communicable diseases are the leading cause of death, accounting for 43.1% of deaths recorded between 2011 and 2015 (see Table 4 and Figure 5). Among these, HIV/AIDS, malaria, p0077neumonia and PTB were found to be the leading causes of death in all age groups (see Table 3). Our results are largely consistent with expectations and with similar studies conducted in rural Kenya, such as the in the KEMRI/CDC-Kisumu HDSS [24]. As anticipated, we found a slight excess of deaths among males (2677; 51.5%) in comparison to females (2519; 48.5%).

HIV/AIDS was the leading cause of death in the study population, accounting for 12.6% of all deaths (see Table 3). The HDSS is located in a region with known high HIV prevalence (15.6%), three times the nationally reported average of 5.1% [14]. In terms of age, the age group 15–49 years bore the highest burden of HIV/AIDS deaths. The rates were, however, slightly lower than those reported in Kisumu HDSS (16.7%) but expectedly higher than those in Nairobi and Kilifi (9.8% and 10.7%, respectively) [25]. The observed difference could be explained by the different study and reporting periods. The Kisumu HDSS reported on data collected during 2001–2012, while this report covers the period from 2011–2015. Increased access to HIV/AIDS care and treatment since 2001 likely contributed to improved HIV outcomes in the study populations, as has been reported elsewhere [26].

Malaria remains a major public health concern, especially among children under the age of five years. Within the Kombewa HDSS population, malaria was the second leading cause of death, accounting for 10.3% of all deaths. Rates of death attributed to malaria were largely consistent with those in the Kisumu HDSS (11.6% of all deaths), and were higher, as expected, than rates of death attributed to malaria in Nairobi and Kilifi (1.1% and 2.8%, respectively) [27]. Kisumu and Kombewa are known to be malaria endemic.

The HDSS platform is very important for health policy evaluation and has many potential applications. For example, the site participated in several multicenter clinical trials of the RTS,S/AS01 malaria vaccine, including the large phase 3 trial in African children in which episodes of both clinical and severe malaria in children receiving the vaccine at 5–17 months of age were reduced by approximately 50% [28]. The RTS,S/ASO1 vaccine is expected to be incorporated into routine immunization schedules soon after it has been licensed and approved for use. The HDSS provides a platform within which to monitor whether deaths attributable to malaria decrease in the years following the introduction of the vaccine.

Of particular interest are external causes of death, such as road traffic accidents, which accounted for 7.4% of all deaths. Road traffic accidents, accounted for 23.5% (68/289) of all external causes of death (see Table 4). This result is consistent with findings from the Kilifi HDSS, which found transport accidents to be the leading cause of external deaths [29]. The high numbers of traffic accidents in Kombewa and Kilifi might be explained by the popularity of motorcycles as a means of transport. The sex differential is expected as the majority of riders are young males, most of whom have no training and wear little or no protective clothing like riding suits and helmets. In the Kisumu HDSS; however, the leading cause of external deaths was suicide [30]. This was unexpected, given that the areas covered by the two HDSS platforms are contiguous, but may be explained by the busy Kisumu–Busia highway which cuts through the eastern part of the Kombewa HDSS. The highway connects Kenya and Uganda, and is a popular route for buses ferrying Kenyan traders and visitors to Uganda as well as trucks transporting fuel to Uganda.

Suicide accounted for 21.1% of the 289 deaths due to injuries in the Kombewa HDSS. These deaths were more common in the 15–49 year age group; similar results were reported in the Kisumu HDSS, Kilifi, and Nairobi. It is unclear what the driving force behind this is, however, drug and alcohol use, stigmatization due to HIV/AIDS, or high unemployment rates may contribute. Assault cases accounted for 16.6% of all external causes of deaths. The age group 15–49 accounted for 68.8% of all deaths due to assault.

NCDS have been shown to be a major contributor of disease burden in the developing world, especially among adults [31]. In the Kombewa HDSS the greatest burden of NCDs was among the elderly (aged 65+ years), accounting for 57.8% of all deaths due to NCD. NCDs accounted for 34.4% of all causes of death in the HDSS. Overall, the leading causes among NCDs were cancers, acute abdomen, and stroke. The top causes of cancer deaths were stomach cancers, reproductive cancers, respiratory cancers, and breast cancer (see Table 4).

## Conclusion

Timely and reliable statistics on deaths are necessary for population health assessment, epidemiological research, health planning, and programme evaluation. Longitudinal data from population-based surveillance, such as a HDSS, provide crucial time-trend age-specific, cause- specific mortality data that can be pivotal in addressing public health concerns and supporting research activities. This dataset provides opportunities for a wide range of more detailed analyses based on the different categories of public health interest such as childhood causes of death, pregnancy related mortality, and NCDs among others.

## Paper context

Computer-based models such as the InterVA-4 are increasingly being relied upon in an attempt to overcome the longstanding limitations of alternative methods of deriving COD data, such as physicians coding. The analysis presented here, establishes the main CODs among people of all ages within the area served by the Kombewa HDSS. We hope this information will help address preventable deaths in the surveyed community and mitigate negative health impacts in other regions.
